# *Ex Vivo* Near-Infrared Molecular Imaging of Human Upper Urinary Tract Urothelial Carcinoma With a CD47-Based Targeted Tracer

**DOI:** 10.3389/fonc.2022.825476

**Published:** 2022-02-28

**Authors:** Pengyu Yan, Dan Chen, Xutao Yan, Xiaoting Yan, Yingpeng Wang, Chao Liu, Xiaofeng Yang

**Affiliations:** ^1^ Department of Urology, First Hospital of Shanxi Medical University, Taiyuan, China; ^2^ Department of Pathology and Cancer Research Center, Yanbian University Medical College, Yanji, China

**Keywords:** upper urinary tract urothelial carcinoma, CD47, near-infrared fluorescence, molecular imaging, targeted imaging

## Abstract

**Objective:**

The low detection rate of early and small tumors remains a clinical problem that urgently needs to be solved in the accurate diagnosis and treatment of upper urinary tract urothelial carcinoma (UTUC). The objective of this study is to evaluate the feasibility of CD47 as a target for optical molecular imaging of human UTUC and conduct preliminary *ex vivo* imaging experiments.

**Methods:**

We firstly analyzed the genome-wide mRNA expression data from Gene Expression Omnibus (GEO). Paraffin-embedded tissue specimens comprising UTUC and normal urothelium were collected. All tissue specimens were used for immunohistochemistry to compare CD47 protein expression in normal and cancer tissue. Meanwhile, 12 patients undergoing radical nephroureterectomy were prospectively included in *ex vivo* imaging experiments. Freshly isolated upper urinary tract specimens were incubated with anti-CD47-Alexa Fluor 790 and then imaged under white light and near-infrared (NIR) light. Standard histopathologic evaluation was performed, and findings were correlated with CD47-targeted NIR molecular imaging.

**Results:**

The GEO data revealed that CD47 mRNA expression was higher in UTUC specimens than that in paracancer normal tissue. In immunohistochemical analysis, the CD47 protein expression level was higher in both non-muscle-invasive and muscle-invasive (stage ≥T2) UTUCs than that in normal uroepithelium, and the localization of CD47 protein was the tumor cell membrane. In the *ex vivo* imaging experiments, all patients were pathologically diagnosed with UTUC, and no adverse effects of anti-CD47-Alexa Fluor 790 on the histological structure of the tumor and normal uroepithelium were observed. In the NIR grayscale images, the mean fluorescence intensity of the tumor tissue was significantly higher than that of the adjacent normal background tissue, which greatly improved the visualization of the tumor.

**Conclusions:**

CD47-targeted NIR molecular imaging could be a feasible and powerful strategy for the accurate diagnosis of UTUC. Larger-scale randomized trials are needed.

## Introduction

Urothelial carcinomas (UCs) rank fourth in the most common tumors, 90%–95% among them occurring in the lower urinary tract (bladder and urethra) (1). Nevertheless, the upper urinary tract urothelial carcinomas (UTUCs) account for merely 5%–10% of all UCs, with an estimated annual incidence rate of 2/100,000 inhabitants in western countries (2). Approximately 60% of newly diagnosed UTUCs are muscle-invasive lesions (stage ≥T2) while only 20%–25% of bladder tumors (3). Radical nephroureterectomy (RNU) plus bladder cuff excision is the standard treatment for UTUC, followed by adjuvant therapy tailored to the individual risk of recurrence and progression. Even after receiving the standard regimens, bladder recurrence occurs in 22%–47% of patients compared with 2%–6% recurrence in the contralateral upper tract, necessitating rigorous follow-up (4, 5).

The European Association of Urology (EAU) 2020 updated guidelines strongly recommend that all low-risk UTUCs (unifocal, low-grade, noninvasive, tumor size <2 cm) should discuss kidney-sparing surgery because it can obtain similar survival as RNU but with fewer complications (e.g., renal insufficiency) (2, 6). Therefore, to improve the chances of survival and nephron-sparing, an important medical goal is to identify early lesions, including carcinoma *in situ* (CIS). Currently, the preferred approach for the diagnosis of UTUC is the computed tomography urography (CTU) due to its high detection rate (2). However, early and small lesions without mass effect or thickening of the urothelium are usually invisible on CT. Ureteroscopy (URS) can be used to visually determine the presence, location, and size of tumors but misses CIS in approximately 50% of cases (7). Undergrading and understaging occur in more than one-third of patients following ureteroscopic biopsy (8).

To improve the detection of early and small malignant lesions, several novel optical imaging techniques have begun to be evaluated *in vivo*, but their current applications still have limitations. Photodynamic diagnosis and narrow-band imaging can improve the detection rate of papillary lesions and CIS, considering their non-tumor-specific nature, inflammation, and acute bleeding will lead to a significant increase in false-positive results (9, 10). Confocal laser endomicroscopy and optical coherence tomography provide pathological information on tumor in real time, but their small field of view makes it difficult to effectively scan the entire uroepithelium to detect lesions missed by URS (11, 12). Optical molecular imaging is a combination of novel optical imaging techniques and modern molecular biology. The binding of targeted fluorescent tracers with overexpressed molecules on tumor cells or tumor microenvironment allows qualitative and quantitative analysis of the biological behavior of tissues at the molecular and cellular levels prior to their macroscopic structural changes (13). With paired optical imaging devices, it is possible to detect small or occult tumors with minimized false-positive results.

CD47 is an innate immune checkpoint expressed in various human solid cancer cells (14). Accumulating studies reveal that cell surface expression of CD47 is a common mechanism by which cancer cells protect themselves from phagocytosis (14). In previous studies, anti-CD47 antibody has been used for targeted imaging and tumor-specific drug delivery in bladder urothelial carcinoma (BUC) (15, 16). UTUC and BUC share the same morphologic and histologic presentation, but molecular data suggest that they are distinct disease entities (17). To scientifically and systematically assess the feasibility of CD47 as a molecular imaging target for UTUC, we first verified the differential expression of CD47 between UTUC and normal uroepithelium at the mRNA and protein levels. Subsequently, we performed *ex vivo* near-infrared (NIR) molecular imaging of 12 freshly isolated UTUC by intraluminal perfusion of the NIR fluorescent dye Alexa Fluor 790-labeled anti-CD47. This investigation provides a basis for the clinical transformation of CD47 as a molecular imaging target of UTUC and provides new ideas for a wider range of diagnosis and treatment options.

## Materials and Methods

### Identification of Differentially Expressed Genes Associated With Upper Urinary Tract Urothelial Carcinoma

Genome-wide mRNA expression spectrum sequencing data of UTUC were downloaded from Gene Expression Omnibus (GEO; https://www.ncbi.nlm.nih.gov/geo/). Two datasets (GSE47702 and GSE134292) were included, containing a total of 90 tumor samples and 10 paracancer normal samples. Gene expression of all samples was normalized to transcripts per million clean tags (TPM), and differentially expressed genes (DEGs) were identified based on the R package *limma*. We identified the genes satisfying the criteria of |log2FC| >1 [fold change (FC)] and *P* < 0.01 to be DEGs.

### Immunohistochemistry

Cases with a pathological diagnosis of UTUC by surgical resection between August 2018 and September 2020 at our center were collected. Excluding cases with high-grade and low-grade coexistence or with histological variants and excluding cases with concomitant bladder cancer, paraffin-embedded specimens and clinical data of 57 tumors and 20 contemporaneous normal uroepithelial cases were finally included. The tissue blocks were sectioned into 4-μm-thick sections, placed on adhesion slides, deparaffinized, rehydrated, heat antigen retrieval, and endogenous peroxidase-inactivated. Incubated with rabbit polyclonal anti-CD47 (1:100, ab175388, Abcam, Cambridge, MA, USA) overnight at 4°C, followed by exposure to horseradish peroxidase (HRP)-labeled IgG (1:500, ab6721, Abcam) for 30 min. Finally, the antibodies were visualized with diaminobenzidine (DAB) and counterstained with hematoxylin. A double-blind method was used to observe the results, and five non-overlapping staining fields were randomly selected for each IHC section at ×400 field of view for observation. The mean optical density (ratio of signal intensity to area) was analyzed with Image Pro Plus 6.0 (Media Cybernetics, Silver Spring, MD, USA) under uniform conditions.

### *Ex Vivo* Near-Infrared Molecular Imaging

Approved by the ethics committee of the host institution. From September 2020 to March 2021, a total of 12 patients who underwent laparoscopic RNU were prospectively included in the study. The patients being studied include primary, unifocal and not the recurrent upper urinary tract tumors, which are proven by URS, CTU, or magnetic resonance urography (MRU). It is worth noting that we did not select the UTUC patients with these properties, who have bladder tumors simultaneously, histories of bladder cancer or contralateral UTUCs, suspected distant metastasis *via* preoperative examinations, or large tumors (diameter >5 cm). All patients were informed and consented to participate in this study, and the procedure was performed by the same experienced urologist.

Fresh and intact upper urinary tract specimens were collected from the operating room immediately after excision. Anti-CD47-Alexa Fluor 790 (excitation, 760 nm; emission, 835 nm; 200 μg/ml; Santa Cruz Biotechnology, Santa Cruz, California, USA) was diluted with phosphate buffer saline at a ratio of 1:100 to prepare 50 ml of imaging probe, which was slowly injected into the specimen lumen through a 6F ureteral catheter. After incubation for 30 min at 37°C, the unbound antibody is removed by three rinses with 100–200 ml of sterile saline. The washed upper urinary tract specimens were incised longitudinally and placed under visible and NIR Fluorescence separated-merged imager (SES Co., Taiyuan, China) for imaging. This imager captured the visible light information and NIR fluorescence information of the UTUC samples in real time. Then displayed the white light image, fluorescence image, and fusion image on the screen simultaneously. Positive anti-CD47-Alexa Fluor 790 fluorescence is defined as the bright area in NIR grayscale image that is independent of shooting angle. The mean fluorescence intensity (MFI) of the corresponding tissues was expressed according to the mean grayscale value of the tumor and the adjacent normal background in the NIR grayscale image. Tumor-to-background ratio (TBR) was calculated as the ratio of MFI of tumor tissue to MFI of normal background tissue. After imaging, the fluorescent areas were marked by ink and entirely submitted for standard histopathological examination by the same pathologist. Histological grades and tumor stages were assessed according to the 2004 WHO grading system and the 2017 TNM system, respectively.

### Statistical Analysis

Quantitative data conforming to normal distribution were described as mean ± standard deviation, and a t-test was used to evaluate the differences. Measures that did not conform to normal distribution were recorded as median (range) and compared through Wilcoxon rank-sum test. Qualitative data were expressed using numbers and percentages and compared by chi-square test or Fisher exact test. The data were analyzed using R 4.0.3 and GraphPad Prism 8.0 for Windows, with *P*-value <0.05 considered statistically significant. All statistical analyses were performed two-tailed.

## Results

### Differential Expression of CD47 mRNA

**Figure 1:**
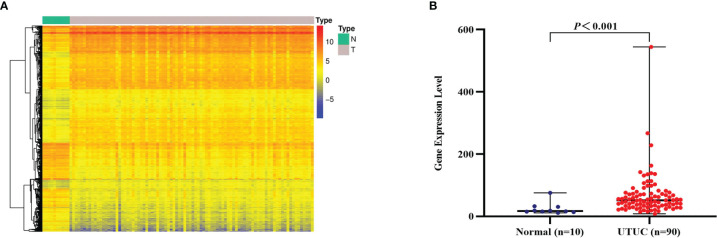
Identification and analysis of differentially expressed genes (DEGs) and CD47 mRNA expression. **(A)** Hierarchical clustering of DEGs, with rows representing genes and columns representing samples. For a gene, red represents a higher expression level, blue represents a lower expression level, and yellow represents the median expression level for all samples. **(B)** CD47 mRNA expression levels in the upper urinary tract urothelial carcinoma (UTUC) (n = 90) group and normal group (n = 10) (|log2FC| = 1.43, *P* < 0.001), and *P*-values were determined using Wilcoxon rank-sum test.

After surveying published papers and preliminary experimental data, we concluded that CD47 may be a viable optical molecular imaging target for UTUC. Thus, we designed a systematic validation scheme. We first analyzed whole-genome transcriptome sequencing data of 90 UTUC samples and 10 paracancer normal samples from the GEO database. With the thresholds |log2FC|>1 and *P* < 0.01, a total of 5,421 DEGs were finally identified from the UTUC and normal groups, of which 2,125 were upregulated and 3,296 were downregulated (**Figure 1A**). We next focused on analyzing the expression of CD47 mRNA in the same dataset. CD47 mRNA expression was markedly higher in the UTUC group compared with that in the normal group(|log2FC| = 1.43, *P* < 0.001) (**Figure 1B**).

### CD47 Protein Expression and Distribution

**Figure 2:**
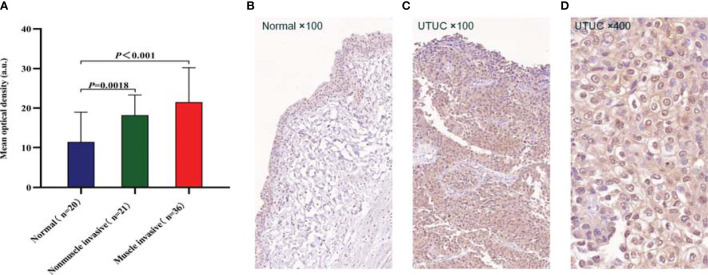
Immunohistochemistry (IHC) for CD47 protein expression and distribution. **(A)** Qualitative analysis of IHC results revealed that CD47 protein expression was higher in both non-muscle-invasive (n = 21) and muscle-invasive (n = 36) upper urinary tract urothelial carcinomas (UTUCs) than that in normal uroepithelium (n = 20) (*P* < 0.01), and *P*-values were determined using unpaired t tests. **(B)** CD47 was expressed in trace amounts in normal uroepithelium. **(C)** CD47 was highly expressed in UTUC tissues, CD47-positive cells were widely distributed in the cancer tissue, including on the luminal surface. **(D)** The localization of CD47 protein was mainly the tumor cell membrane.

To further confirm the expression level and distribution characteristics of CD47 protein, we performed immunohistochemical staining on formalin-fixed paraffin-embedded sections of 57 tumors and 20 contemporaneous normal urothelium from our center. Clinical information and pathologic information of patients are shown in **Table 1**. The results showed that the expression level of CD47 was higher in both non-muscle-invasive tumors (n = 21) and muscle-invasive tumors (n = 36) than in normal uroepithelium (n = 20); the mean optical densities were 18.21 ± 5.10, 21.49 ± 8.73, and 11.44 ± 7.51, respectively (*P* < 0.01; **Figure 2A**). We also found that the localization of CD47 protein was mainly the tumor cell membrane, whereas CD47 was widely distributed in the cancer tissue including on the luminal surface (**Figures 2B–D**).

**Table 1:** Clinical and pathologic information of patients with immunohistochemical staining.

Variable	UTUC (n = 57)	Normal (n = 20)	*P*
Age (years)	66 (52–83)	64 (50–83)	0.51
Gender, n (%)			0.28
Male	34 (59.65%)	15 (75.00%)	
Female	23 (40.35%)	5 (25.00%)	
Tumor diameter (cm)	3.0 (0.5–11.0)	–	
Side			0.99
Left	30 (52.63%)	10 (50.00%)	
Right	27 (47.37%)	10 (50.00%)	
Tumor location, n (%)			0.81
Renal pelvis	34 (59.65%)	11 (55.00%)	
Upper ureter	10 (17.54%)	3 (15.00%)	
Lower ureter	13 (22.81%)	6 (30.00%)	
Grade (WHO 2004), n (%)			–
LG	19 (33.33%)	–	
HG	38 (66.67%)	–	
T stage, n (%)			–
pTa	13 (22.81%)	–	
pT1	9 (15.79%)	–	
pT2	17 (29.82%)	–	
pT3	14 (24.56%)	–	
pT4	4 (7.02%)	–	

WHO, World Health Organization; LG, low grade; HG, high grade; -, no data.

### CD47-Targeted Near-Infrared Molecular Imaging of Freshly Isolated Upper Urinary Tract Urothelial Carcinoma

**Figure 3:**
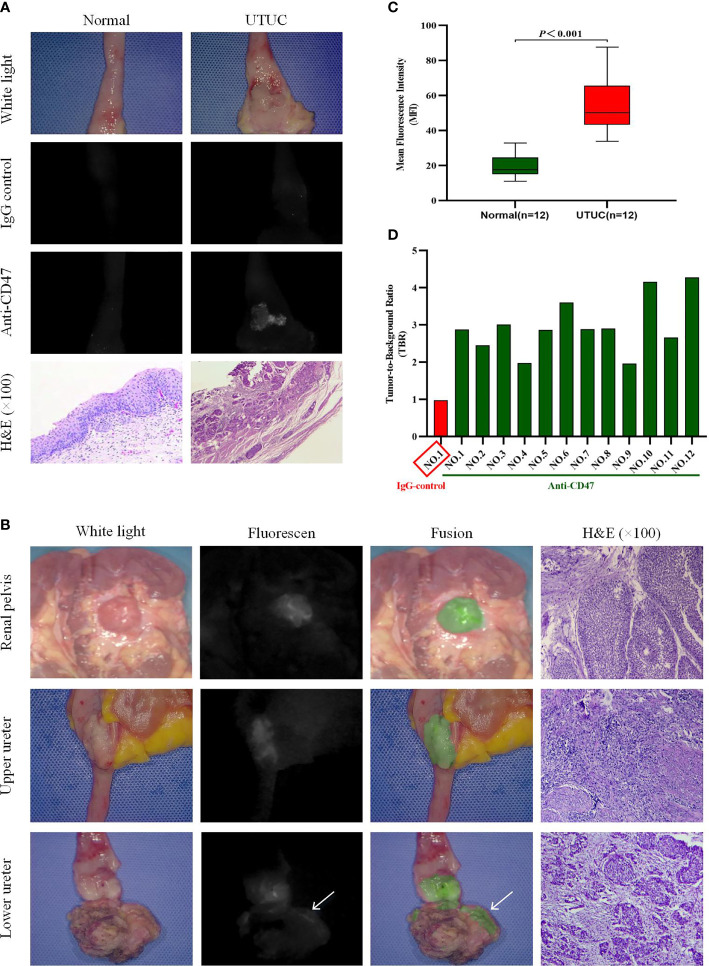
Near-infrared (NIR) molecular imaging of freshly isolated human upper urinary tract urothelial carcinoma (UTUC) with anti-CD47-Alexa Fluor 790. Representative white-light, NIR-light, and fusion images with the relevant hematoxylin and eosin-stained (H&E, ×100) photomicrographs for colocalization of anti-CD47-Alexa Fluor 790 binding and histopathology. **(A)** One upper urinary tract specimen was incubated and imaged successively with IgG control-Alexa Fluor 790 and anti-CD47-Alexa Fluor 790. White- and NIR-light images of normal urothelium and a noninvasive papillary urothelial carcinoma after incubation with the two antibodies are shown. **(B)** Representative images of renal pelvis, upper ureter, and lower ureter UTUC after incubation with anti-CD47-Alexa Fluor 790. Positive fluorescence at the surgical margin (at the arrow) was seen in the lower ureteral tumor. **(C)** Quantitative mean fluorescence intensity (MFI) of 12 specimens. MFIs of tumor tissue and normal tissue were 54.67 ± 16.17 and 19.11 ± 6.41 (*P* < 0.001), respectively. **(D)** Tumor-to-background ratio (TBR) data of each patient.

In order to confirm that the fluorescence detected on the cancer is the result of specific binding to CD47, we first incubated one sample with isotype immunoglobulin G (IgG)-Alexa Fluor 790 as a negative control. As expected, both tumor tissue and normal uroepithelium showed only low levels of background fluorescence intensity (MFIs are respectively 14.15 and 14.38). Subsequently, we reincubated the uroepithelium of the same sample and the tumor with anti-CD47-Alexa Fluor 790, rinsed with sterile saline, and imaged again. Under NIR light, a 2.94-fold increase in fluorescence signal was detected at the tumor lesion, while no significant change in fluorescence was observed in the normal surrounding tissue (MFIs are respectively 44.08 and 15.31). The corresponding histopathology of the lesion with significantly elevated MFI confirmed noninvasive papillary urothelial carcinoma, whereas the area without anti-CD47-Alexa Fluor 790 fluorescence confirmed normal urothelium (**Figure 3A**).

We performed *ex vivo* CD47-targeted NIR molecular imaging on a total of 12 samples. Histopathological examination confirmed that all 12 samples had UTUC, and no adverse effects of anti-CD47-Alexa Fluor 790 on tumor or normal uroepithelial histology were seen. **Table 2** includes the patient’s demographic data, histopathological diagnosis, and molecular imaging results. Representative imaging results of tumors located in the renal pelvis, upper ureter, and lower ureter are shown in **Figure 3B**. And we found that the contrast of visual image was evidently enhanced under the optical molecular imaging technology that could help doctors improve the visual effect of tumor. Notably, in this case of lower ureteral tumor, NIR molecular imaging revealed a significant fluorescent at the distal margin, which was later pathologically confirmed to be a positive surgical margin. Overall, qualitative assessment of fluorescence signals in all patients showed higher MFI in tumor tissue compared to that of normal background tissue with NIR optical molecular imaging techniques (54.67 ± 16.17 vs. 19.11 ± 6.41, *P* < 0.001) (**Figure 3C**). TBR of fresh isolated UTUC specimens ranged from 1.7 to 4.09 (**Figure 3D**). Although the TBR was higher in muscle-invasive lesions than that in non-muscle-invasive lesions (3.05 ± 0.89 vs. 2.80 ± 0.24), the difference was not statistically significant (*P* = 0.601).

**Table 2:** Patient’s demographic data, histopathological diagnosis, and molecular imaging results with anti-CD47-Alexa Fluor 790 NIR molecular imaging.

Case no.	Gender/Age (years)	Tumor diameter (cm)	Side/location	Grade (WHO 2004)	T stage	Tumor MFI/Background MFI	TBR
1	Female/68	1.0	Left/Lower ureter	LG	pTa	44.08/15.31	2.88
2	Male/63	2.5	Right/Renal pelvis	LG	pT1	64.62/26.38	2.45
3	Female/44	4.5	Left/Renal pelvis	HG	pTa	55.82/18.55	3.01
4	Male/62	3.0	Left/Upper ureter	LG	pT3	33.92/17.15	1.98
5	Female/75	1.1	Left/Lower ureter	HG	pTa	43.23/15.08	2.87
6	Female/80	2.0	Right/Upper ureter	HG	pT3	66.15/18.38	3.60
7	Male/65	2.2	Left/Lower ureter	HG	pT3	44.69/15.46	2.89
8	Female/56	2.5	Left/Upper ureter	LG	pT2	76.62/26.46	2.90
9	Male/72	1.6	Left/Lower ureter	LG	pT3	38.77/19.77	1.96
10	Female/69	2.8	Left/Renal pelvis	HG	pT3	53.08/12.77	4.16
11	Female/65	4.7	Left/Renal pelvis	LG	pT3	87.54/32.92	2.66
12	Male/64	0.9	Right/Lower ureter	HG	pT2	47.46/11.08	4.28

WHO, World Health Organization; MFI, mean fluorescence intensity; TBR, tumor-to-background ratio; LG, low grade; HG, high grade.

## Discussion

Tumor grade and stage are the recognized prognostic factors for UTUC (18). Therefore, early and accurate diagnosis is a key factor in defining treatment and improving survival. CTU has the highest detection rate among the noninvasive techniques in UTUC. A recent systematic review comprising 1,233 patients revealed that the pooled sensitivity and specificity of CTU for UTUC were 92% (CI 85%–96%) and 95% (CI 88%–98%), respectively (19). However, CTU often cannot distinguish between small and flat solid tumors nor can it identify chronic inflammation. With the development of endourologic technologies, flexible ureteroscopy (fURS) has been increasingly used to biopsy and diagnose suspicious UTUCs. Grahn et al. (20) investigated 174 renal units in 148 patients and found that fURS had significantly higher accuracy and specificity than CTU despite similar sensitivities. Although many methods, such as CTU, MRU, and fURS, exist for detecting UTUC, 60% of newly diagnosed UTUCs are muscle-invasive tumors compared with 20%–25% of bladder tumors (3). Survival outcomes of invasive UTUCs tend to be poor, with 5-year specific survival rates less than 50% for pT2/pT3 and less than 10% for pT4 (21–24).

Recognizing the need for better and more accurate diagnostic methods, a number of novel optical imaging techniques have been proposed. Depending on their field of view, these techniques can be divided into macroscopic and microscopic imaging modalities. Macroscopic imaging tools as photodynamic diagnosis (PDD) and narrow-band imaging (NBI) aim at better visualization of suspicious malignant lesions with additional image enhancement and investigate large areas of uroepithelium in a similar manner to plain white light URS. Due to their non-tumor-specific nature, although PDD and NBI increase the additional detection of UTUC, particularly for CIS, false-positive results may occur in previously treated luminal, inflammatory lesions and acute bleeding (9, 10). Microscopic imaging tools as confocal laser endomicroscopy (CLE) and optical coherence tomography (OCT) are also known as optical biopsies, providing real-time pathological information by generating high-resolution images of tissue *in vivo* (11, 12). Due to their small field of view, the probe needs to be *en face* contact with the tissue of interest during imaging. Therefore, a combination of other macroscopic imaging techniques (e.g., PDD, NBI, or URS) is required to localize suspicious tissue before using microscopic imaging modalities. Using the optical molecular imaging (OMI) technology, the region of interest such as the tumor tissue could be presented precisely with the function of the specific binding between molecular fluorescent tracer and target. Therefore, OMI can specifically show the pathological process related to tumorigenesis and provide real-time macroscopic imaging of tumor tissue to decrease the incidence of false-positive results. Multiple studies have revealed that it could be used to improve the quality of disease management, such as detection of small or occult tumor lesions and evaluation of the status of surgical margin during tumor resection (13).

Acidity is considered a generic property of the tumor microenvironment due to increased metabolism (25). The pH low insertion peptide (pHLIP) can specifically target acidic cells by inserting into the cell membrane when extracellular pH is low. Brito et al. (26) performed NIR molecular imaging of isolated upper urinary tract specimens from 12 patients using an indocyanine green-coupled pHLIP (ICG-pHLIP), and pHLIP-mediated NIR molecular imaging detected more tumor lesions compared to ordinary white light examination (detection rate 78.9% vs. 100%). Golijanin et al. (27) performed targeted imaging by intravesical infusion of ICG-pHLIP in 22 fresh intact bladder specimens after radical surgery, and its sensitivity in diagnosing bladder cancer was 97%; specificity was 100%. But if the targeting of necrotic tissue from previous transurethral resection or chemotherapy was considered as false positive, the specificity drops to 80% (27). Therefore, considering the heterogeneity of UTUC, the identification of more molecular markers capable of classifying different tumor characteristics may help to develop more precise and personalized detection protocols.

CD47 is a member of the immunoglobulin superfamily; it is believed to be able to bind to the signal regulatory protein α on the surface of macrophages and transmitting a “don’t eat me” signal, causing immune escape from tumors (14). Blockade of CD47 by targeted monoclonal antibodies enabled macrophage engulfment of bladder cancer cells *in vitro* and inhibited tumor growth and increased the survival of mouse xenotransplantation models (16, 28). CD47 is expressed on more than 80% of bladder cancer cells but cannot be detected in normal urothelium and the superficial umbrella cell (15). In this context, we demonstrated for the first time that CD47 is highly expressed in UTUC at mRNA and protein level, providing a theoretical basis for the following cancer-specific targeted imaging. In our study, Alexa Fluor 790 was used to label anti-CD47 to construct the targeted tracer. Alexa dyes and their conjugates are more fluorescent and more photostable than their commonly used spectral analogs such as Cy3, rhodamine B, and Texas Red (29). *Ex vivo* molecular imaging of 12 UTUC cases showed that fluorescent tracers based on anti-CD47 targeting guidance specifically bind to CD47 molecules on the surface of tumor cells. Under NIR light, the fluorescence intensity of UTUC and normal urothelium is significantly different (MFI 54.67 ± 16.17 vs. 19.11 ± 6.41, *P* < 0.001), which is helpful for real-time diagnosis of tumors. Molecular imaging also successfully identified a positive surgical margin for a tumor of the lower ureter in our cohort. A multicenter analysis of 472 UTUC patients who underwent open RNU showed that positive surgical margins were an independent predictor of poorer metastasis-free survival (hazard ratio, 2.7; *P* = 0.001) (30). In the future, CD47-based optical molecular imaging could help surgeons make initial judgments about whether tumor tissue in fresh specimens has positive surgical margins or even be used for fluorescence-guided surgery to improve the thoroughness of tumor resection.

To our knowledge, this is the first publication of a protein molecule that can be used for UTUC-targeted imaging. Despite the promising results, there are still many limitations in this study. First, this is a small *ex vivo* feasibility study, and we have strictly selected the included patients. The results of the current study still need to be further validated in a larger cohort. Second, more pathological types need to be included in further studies, such as UCs with squamous differentiation, adenocarcinoma, and heterogeneous hyperplasia. However, due to the low incidence of UTUC, these pathological types may be uncommon and therefore require longer specimen collection time. Finally, if clinical translation is considered, a human-available targeted tracer also needs to be established. The CD47 antibody B6H12 we use is a murine monoclonal antibody, which may not be safe for human use. However, a humanized monoclonal CD47 antibody, HU5F9-G4, is in phase 1 clinical trials for therapeutic use in advanced solid tumors and lymphomas (31). Preliminary results indicate that most toxic reactions are mild to moderate, although adverse reactions such as transient anemia (57%) and peripheral blood smear hemagglutination (36%) may occur (31). For clinical translation of Alexa Fluor 790-labeled CD47 antibodies, additional pharmacology and toxicology studies will be performed to examine the safety prior to human trials. Even if this tracer cannot be administered systemically, intrathecal instillation may prove a safe alternative.

## Conclusions

In this study, we confirmed the high expression of CD47 in human UTUC at the gene and protein levels; meanwhile, we validated the feasibility of this new molecular target by incubating the fresh isolated UTUC specimens with the molecular fluorescent tracer of anti-CD47-Alexa Fluor 790. It is also worth pointing out that the contrast of visual image was evidently enhanced under the CD47-targeted NIR molecular imaging, which is helpful for real-time diagnosis of UTUC. In general, CD47-based NIR molecular imaging is a promising new diagnostic method, but to achieve perfect clinical applications still requires multidisciplinary collaboration between urologists, chemists, physicists, and pharmacologists.

## Data Availability Statement

The datasets presented in this study can be found in online repositories. The names of the repository/repositories and accession number(s) can be found below: https://www.ncbi.nlm.nih.gov/geo/, GSE47702 and GSE134292.

## Ethics Statement

The studies involving human participants were reviewed and approved by the Ethics Committee of First Hospital of Shanxi Medical University (approval ID: K073/2020). The patients/participants provided their written informed consent to participate in this study.

## Author Contributions

(I) Conception and design: PY, CL, and XFY. (II) Administrative support: CL and XFY. (III) Provision of study materials or patients: XTY and XFY. (IV) Collection and assembly of data: PY and DC. (V) Data analysis and interpretation: PY, XY, and YW. (VI) Article writing: All authors. (VII) Final approval of article: All authors.

## Funding

This research was supported by the Central Guidance on Local Science and Technology Development Fund of Shanxi Province (No. YDZJSX2021C010) and the Natural Science Foundation of Shanxi province (No. 20210302124590). The funder had no role in study design, data collection and analysis, decision to publish, or preparation of the article.

## Conflict of Interest

The authors declare that the research was conducted in the absence of any commercial or financial relationships that could be construed as a potential conflict of interest.

## Publisher’s Note

All claims expressed in this article are solely those of the authors and do not necessarily represent those of their affiliated organizations, or those of the publisher, the editors and the reviewers. Any product that may be evaluated in this article, or claim that may be made by its manufacturer, is not guaranteed or endorsed by the publisher.

## Abbreviations

UCs, urothelial carcinomas; UTUCs, upper urinary tract urothelial carcinomas; RNU, radical nephroureterectomy; EAU, European Association of Urology; CIS, carcinoma *in situ*; CTU, computed tomography urography; URS, ureteroscopy; BUC, bladder urothelial carcinoma; NIR, near-infrared; DEGs, differentially expressed genes; GEO, Gene Expression Omnibus; IHC, immunohistochemistry; MRU, magnetic resonance urography; MFI, mean fluorescence intensity; TBR, tumor-to-background ratio; WHO, World Health Organization; TNM, tumor, node, metastasis; PDD, photodynamic diagnosis; NBI, narrow-band imaging; CLE, confocal laser endomicroscopy; OCT, optical coherence tomography; pHLIP, pH low insertion peptide.
